# Risk of Ischemic Stroke Associated with the Use of Antipsychotic Drugs in Elderly Patients: A Retrospective Cohort Study in Korea

**DOI:** 10.1371/journal.pone.0119931

**Published:** 2015-03-19

**Authors:** Ju-Young Shin, Nam-Kyong Choi, Joongyub Lee, Jong-Mi Seong, Mi-Ju Park, Shin Haeng Lee, Byung-Joo Park

**Affiliations:** 1 Korea Institute of Drug Safety and Risk Management, Seoul, Republic of Korea; 2 Medical Research Collaborating Center, Seoul National University College of Medicine/ Seoul National University Hospital, Seoul, Republic of Korea; 3 Department of Preventive Medicine, Seoul National University College of Medicine, Seoul, Republic of Korea; San Francisco, UNITED STATES

## Abstract

**Objective:**

Strong concerns have been raised about whether the risk of ischemic stroke differs between conventional antipsychotics (CAPs) and atypical antipsychotics (AAPs). This study compared the risk of ischemic stroke in elderly patients taking CAPs and AAPs.

**Method:**

We conducted a retrospective cohort study of 71,584 elderly patients who were newly prescribed the CAPs (haloperidol or chlorpromazine) and those prescribed the AAPs (risperidone, quetiapine, or olanzapine). We used the National Claims Database from the Health Insurance Review and Assessment Service (HIRA) from January 1, 2006 to December 31, 2009. Incident cases for ischemic stroke (ICD-10, I63) were identified. The hazard ratios (HR) for AAPs, CAPs, and for each antipsychotic were calculated using multivariable Cox regression models, with risperidone as a reference.

**Results:**

Among a total of 71,584 patients, 24,668 patients were on risperidone, 15,860 patients on quetiapine, 3,888 patients on olanzapine, 19,564 patients on haloperidol, and 7,604 patients on chlorpromazine. A substantially higher risk was observed with chlorpromazine (HR = 3.47, 95% CI, 1.97–5.38), which was followed by haloperidol (HR = 2.43, 95% CI, 1.18–3.14), quetiapine (HR = 1.23, 95% CI, 0.78–2.12), and olanzapine (HR = 1.12, 95% CI, 0.59–2.75). Patients who were prescribed chlorpromazine for longer than 150 days showed a higher risk (HR = 3.60, 95% CI, 1.83–6.02) than those who took it for a shorter period of time.

**Conclusions:**

A much greater risk of ischemic stroke was observed in patients who used chlorpromazine and haloperidol compared to risperidone. The evidence suggested that there is a strong need to exercise caution while prescribing these agents to the elderly in light of severe adverse events with atypical antipsychotics.

## Introduction

Conventional antipsychotics (CAPs), older drugs available since the 1960s, have been widely used for decades in the treatment of schizophrenia, acute mania, bipolar disorder, behavioral and psychological symptoms of dementia (BPSD), and delirium. Atypical antipsychotics (AAPs), which are newer drugs developed in the 1990s, are effective for both positive and negative psychotic symptoms, including extrapyramidal symptoms, hypotension, and anticholinergic-like syndromes [1, 2]. Several different types of adverse events have been associated with atypical antipsychotics such as tardive dyskinesia, neuroleptic malignant syndrome, hyperglycemia/diabetes, etc. [3]. The warnings were issued by the Canadian and US FDAs for their increased risk of cerebrovascular adverse events (CVAEs) based on clinical trials with elderly demented patients [4, 5]. Also in 2004, the UK Committee for the Safety of Medicines (CSM) recommended not using risperidone and olanzapine in older patients with dementia, due to a three-fold increase of the risk of stroke [6].

However, the fact that warnings were issued only for AAPs and not for CAPs does not mean that the older drugs are safer, but clinical and epidemiological data was lacking for the older drugs. A systematic review performed in 2010 concluded that it is necessary to compare the risk among antipsychotics [7]. It showed that most randomized controlled trials did not directly compare the safety of each individual antipsychotic or were not sufficiently powered to permit conclusions about any differences found. Observational studies also showed clinical uncertainty and conflicting findings about this risk. In addition, the majority of previous studies grouped drugs together as conventional or atypical, but the two groups were composed of individual drugs with distinct chemical and biological profiles. Most previous studies have pooled outcomes as both ischemic and hemorrhagic stroke grouped together and have suggested a higher risk of stroke with atypical antipsychotics than with conventional antipsychotics [8–10]; however, ischemic and hemorrhagic stroke are different, with distinct mechanisms. The study by Gill et al. reported that atypical antipsychotics are associated with a similar risk of ischemic stroke as compared with conventional antipsychotics (HR 1.01, 95% CI 0.81 to 1.26) [11].

In light of these events and expanding evidence base, we conducted a retrospective cohort study to compare the risk of hospitalization for ischemic stroke among elderly patients taking antipsychotics. We directly evaluated the risk of ischemic stroke with dose-response, comorbidities, and concurrent medications with adjustment for the estimated propensity scores.

## Methods

### Data Source

The Korean Health Insurance Review and Assessment Service (HIRA) database was used for this study. The National Health Insurance (NHI) program was initiated in Korea in 1977 and achieved universal coverage by 1989 [12]. All Koreans are covered by the National Health Insurance System. Accordingly, the HIRA database contains all information on healthcare utilization and prescribed medications for approximately 50 million Koreans.

The claims data for elderly patients (aged 65 years and above) who were prescribed at least one antipsychotic medication that had been submitted by healthcare providers from January 1, 2005 through December 31, 2009 were obtained. All potential identifiers in the claims data were removed by HIRA and the information was linked with a new unidentifiable code representing each individual patient. The database included age, gender, diagnosis, ambulatory care utilization at clinics and/or hospitals, hospitalization at hospitals, visit dates, health outcomes such as death dates, types of medical care facilities, regions, and specialties. In addition, the prescribed drug information included the brand name, generic name, prescription date, duration, dose, and route of administration [13]. The diagnoses were coded according to the International Classification of Diseases, Tenth Revision (ICD-10).

### Study population

The cohort consisted of elderly patients who had been newly prescribed AAPs or CAPs between January 1, 2006 and December 31, 2009. Inclusion criteria were the following: (i) patients aged 65 years and above who visited at least one hospital or clinic located in Korea and (ii) patients with no history of taking antipsychotic medications in the 12 months prior to the study entry date. Exclusion criteria were (i) patients with prior cerebrovascular diseases (I60-I69) or TIA (G45) during the prior 365 days, (ii) patients with a diagnosis of ischemic stroke recorded on the first day of antipsychotic prescription, (iii) patients older than 100 years old, and (iv) patients who received non-oral antipsychotics such as injectable or depot preparation.

### Exposure Assessment

Exposure was considered to begin on the date of the first antipsychotic prescription following the eligibility period. The index date was the first prescription date of antipsychotic initiation. To calculate the duration of exposure based on an individual prescription, the number of days’ supply of the medication was added to the date of the antipsychotic prescription. In the event of a prescription before exhausting the previous supply, it was assumed that the patient begin using their new supply on the date of the new prescription. Subjects were considered exposed until the end of the continuous exposure period. A 60-day grace period between periods of antipsychotic exposure was allowed before assuming that the medication was discontinued (S1 Table). Subjects switching antipsychotics or discontinuing antipsychotic therapy were censored at the date of switching or discontinuation. To calculate the mean antipsychotic prescribed daily dose (PDD) for each individual, we multiplied the quantity of pills prescribed by the tablet strength and divided by the number of days’ supply. The most commonly used medications worldwide were selected to represent the conventional antipsychotic and atypical antipsychotic groups. The CAPs included chlorpromazine and haloperidol and AAPs included risperidone, quetiapine, and olanzapine. Among many CAPs, not only were chlorpromazine and haloperidol the top two antipsychotics prescribed, but also a number of additional studies were conducted with haloperidol.

### Ischemic Stroke

The study subjects were observed until their first hospitalization with ischemic stroke (I63). The patients were censored upon the date of the first of the following events: the patient switched to another antipsychotic, the patient discontinued anti-psychotics, the patient died, or the last day of the study. A validation study compared the diagnoses derived from the HIRA database with the actual diagnoses in the patient medical records. The overall positive predictive value of the diagnoses was 83.4% for the hospitalized patients [14–16].

### Potential Confounders

We defined the characteristics of the patients during the year before each subject’s index date according to age, gender, psychiatric characteristics, comorbidities, and comedications. Psychiatric characteristics were determined by ICD-10 codes for the following conditions: dementia, schizophrenia, manic episodes, other psychotic disorders, depressive episodes, bipolar affective disorder, obsessive-compulsive disorder, and delirium. A list of comorbidities and comedications that were potential confounders was compiled. It was based on the previous stroke guidelines, including all well-documented and modifiable risk factors [17]. Comorbidities included hypertension, coronary heart disease, heart failure, atrial fibrillation, other arrhythmias, diabetes mellitus, dyslipidemia, COPD, pneumonia, Parkinson’s disease, coagulopathy, valvular heart disease, thyrotoxicosis, and acute myocardial infarction. The diagnosis of COPD was selected as a proxy for smoking in the elderly. The Charlson comorbidity score was calculated in the year before the index date [18, 19]. Comedications included the use of lithium, mood stabilizers, antidepressants, benzodiazepines, hormone replacement therapy, anticoagulants, antiplatelet agents, warfarin, and antithrombotic agents. Among comedications in the final model, anticoagulants and antithrombotics are chronic medications which are not time varying. Also for benzodiazepines and antidepressants, several studies have shown no time-varying effect [15, 20].

### Statistical Analysis

We calculated distributions of demographic and clinical characteristics and the use of each antipsychotic. Continuous variables were compared with the t-test, and categorical variables were compared with the Mantel-Haenszel chi-squared test. The incidence rates and 95% confidence intervals (CI) of the study outcomes were calculated for each study group. The cumulative hazard rate was also estimated using the Kaplan-Meier method and plotted according to the time. Censored patients were not included in the “number of at risk” at each time and cumulative events were distributed. The probability of an event, probability of survival (= 1-probablitiy of event), survival function, and its cumulative hazard rate (= 1-survival function) were calculated accordingly. The log minus log survival (LML) plot was graphed to test whether the Cox proportional hazards assumption was met. However, the LML plot did not result in parallel curves, which means the Cox proportional hazards assumption is not met. The cross-points estimated using the time-proportionality curve were 150 days for olanzapine, haloperidol, and chlorpromazine and 90 days for quetiapine. Cox regression models were used to estimate the hazard ratio (HR) and 95% confidence intervals (95% CIs) for the event associated with each exposure according to the time interval before and after the cross-point. The results in the models weighted for the propensity score (standardized mortality ratio (SMR) weighted) and multivariable adjusted HR were accepted as the final value after the propensity score restrictor [21–23].

First of all, the unadjusted HRs were evaluated. To adjust for group differences, a propensity score analysis was carried out to control for selection biases and to determine the effect of the exposure to each drug on ischemic stroke. The propensity scores were estimated without regard to outcomes by multiple logistic regression analysis. We constructed three separate logistic regression models to estimate the propensity scores. According to Cadarette et al. [21], calculation of propensity scores with three separate logistic regressions may be used to replace multinomial logistic regression because the asymptotic relative efficiencies of predicted probabilities are generally high. This is also consistent with previous research [22, 23]. A full nonparsimonious model was developed that included all the variables shown in Table 1. Model discrimination was assessed with c statistics, and model calibration was assessed with Hosmer-Lemeshow statistics [24, 25]. Secondly, to construct a propensity score-adjusted model, the individual propensity score was incorporated into the Cox regression model as a covariate of drug exposure.

**Table 1:** General characteristics of new users of conventional and atypical antipsychotic medications.

Characteristics	Atypical Antipsychotics			Typical Antipsychotics		p value*
	**Risperidone**	**Quetiapine**	**Olanzapine**	**Haloperidol**	**Chlorpromazine**	
	**(N = 24,668)**	**(N = 15,860)**	**(N = 3,888)**	**(N = 19,564)**	**(N = 7,604)**	
**Mean Age (y)**	76.2 (7.0)	74.7 (6.7)	73.4 (6.3)	75.3 (6.9)	71.6 (5.6)	<.01†
**Gender, Female (%)**	15,861 (64.3)	9,186 (57.9)	2,444 (62.9)	12,309 (62.9)	2,221 (29.2)	<.01
**Charlson Comorbidity Index (mean, SD)**	1.9 (2.1)	2.4 (2.5)	2.1 (2.1)	2.0 (2.2)	2.4 (2.6)	<.01†
The presence of dementia						
(F00-F03, G30, G31.8)						
Yes	7,228 (29.3)	3,388 (21.4)	697 (17.9)	4,078 (20.8)	648 (8.5)	<.01
**Psychiatric characteristics**						
Schizophrenia (F20)	984 (4.0)	819 (5.2)	321 (8.3)	454 (2.3)	252 (3.3)	<.01
Manic episode (F30)	49 (0.2)	46 (0.3)	17 (0.4)	36 (0.2)	15 (0.2)	0.64
Other psychotic disorders (F22.0, F23, F29)	346 (1.4)	213 (1.3)	112 (2.9)	153 (0.8)	35 (0.5)	<.01
Depressive episode(F32–33, F34.1, 41.2)	4,341 (17.6)	4,306 (27.2)	1,273 (32.7)	3,341 (17.1)	1,089 (14.3)	<.01
Bipolar affective disorder (F31)	518 (2.1)	776 (4.9)	262 (6,7)	239 (1.2)	139 (1.8)	<.01
Obsessive-compulsive disorder (F42)	52 (0.2)	38 (0.2)	27 (0.7)	39 (0.2)	17 (0.2)	0.81
Delirium (F05)	423 (1.7)	304 (1.9)	46 (1.2)	246(1.3)	33 (0.4)	<.01
**Comorbidity affecting risk of stroke**						
Essential Hypertension (I10.0-I15.9)	1,965 (8.0)	1,793 (11.3)	332 (8.5)	1,619 (8.3)	595 (7.8)	0.04
History of Coronary Heart Disease (I21-I25)	12,051 (48.9)	8,843 (55.8)	2,003 (51.5)	9,844 (50.3)	3,620 (47.6)	0.03
Heart Failure (I50)	1,694 (6.9)	1,189 (7.5)	230 (5.9)	1,396 (7.2)	350 (4.6)	<.01
Atrial Fibrillation (I48)	761 (3.1)	690 (4.4)	132 (3.4)	573 (2.9)	207 (2.7)	<.01
Other Arrhythmias (I44.0-I49.9)	1,472 (6.0)	1,352 (8.5)	283 (7.3)	1,214 (6.2)	466 (6.1)	0.24
Diabetes Mellitus (E10-E14)	6,218 (25.2)	5,253 (33.1)	1,102 (28.3)	5,214 (26.7)	2,205 (29.0)	0.01
Dyslipidemia (E78.0)	4,388 (17.8)	4,354 (27.5)	940 (24.2)	3,830 (19.6)	1,606 (21.1)	0.03
Chronic Obstructive Pulmonary Disease(J40-J44, J47)	5,484 (22.23)	3,915 (24.7)	912 (23.5)	4,837 (24.7)	2,010 (26.4)	<.01
Pneumonia (J12-J16)	2,312 (9.4)	1,781 (11.2)	372 (9.6)	1,922 (9.8)	759 (10.0)	0.54
Parkinson disease (G20)	932 (3.8)	1,270 (8.0)	189 (4.9)	668 (3.4)	123 (1.6)	<.01
Coagulopathy (D65-D68)	107 (0.4)	176 (1.1)	18 (0.5)	92 (0.5)	59 (0.8)	0.69
Valvular heart disease (I06–08)	94 (0.4)	73 (0.5)	14 (0.4)	62 (0.3)	18 (0.2)	0.03
Thyrotoxicosis (E05)	484 (2.0)	491 (3.1)	97 (2.5)	372 (1.9)	146 (1.9)	0.04
Acute myocardial infarction (I21)	528 (2.1)	539 (3.4)	85 (2.2)	437 (2.2)	167 (2.2)	0.20
**Concurrent use of medication**						
Lithium	161 (0.7)	146 (0.9)	67 (1.7)	104 (0.5)	98 (1.3)	0.03
Mood stabilizers	3,421 (13.9)	2,935 (18.5)	733 (18.9)	2,958 (15.1)	1,115 (14.7)	0.27
Antidepressants	7,196 (29.2)	6,189 (39.0)	1,729 (44.5)	5,726 (29.3)	1,905 (25.1)	<.01
Benzodiazepine	12,943 (52.5)	9,294 (58.6)	2,531 (65.2)	11,198 (57.2)	4,111 (54.1)	<.01
Hormone replacement therapy	563 (2.3)	449 (2.8)	171 (4.4)	432 (2.2)	144 (1.9)	0.07
Anticoagulants	786 (3.2)	1,025 (6.5)	194 (5.0)	701 (3.6)	395 (5.2)	0.01
Antiplatelet agents	7,473 (30.3)	5,959 (37.6)	1,266 (32.6)	6,104 (31.2)	2,299 (30.2)	0.03
Warfarin	332 (1.4)	413 (2.6)	58 (1.5)	268 (1.4)	101 (1.3)	0.03
Antithrombotic agents	58 (0.2)	52 (0.3)	9 (0.2)	35 (0.2)	24 (0.3)	0.63

* The P value was calculated by using the Mantel-Haenszel chi-squared test.

† The P value was calculated by using an ANOVA test with the Bonferroni correction.

To limit confounding, we looked into the distribution of propensity scores and determined the cut-offs for each antipsychotic. *Exclusion of patients with a propensity score of > 99*.*99 with quetiapine*, *< 0*.*05 with olanzapine*, *> 0*.*90 with haloperidol*, *and < 0*.*05 with chlorpromazine yielded our final estimated risk*. A similar method was applied in a previously published study by Kurth et al.[26]

In addition, for more rigorous adjustment to avoid selection bias, a third Cox model was created with the SMR-weighted method, which had significant effects on the outcome. A SMR-weighted hazard ratio was calculated using weights of one for risperidone users and the odds of the propensity score (PS) for olanzapine, quetiapine, and haloperidol users [PS/(1−PS)][27]. The SMR-weighted method estimates the treatment effect in a population whose distribution is equal to that found in risperidone users. This weighting method is beneficial because it may avoid underestimation related to the selection of a lower-risk population by propensity score matching. Particularly, the SMR method minimizes the possibility of overestimating the risk due to residual confounding resulting from selecting the general population as reference group by inverse-probability-of-treatment weighting (IPTW), which was used in recent analyses of a claims database [26, 28]. Therefore, the SMR-weighted method is more suitable for a comparative effectiveness and safety study.

Lastly, SMR weighted and multivariate adjusted models were used. In this model, the patients whose propensity score was > 99.99 with quetiapine, < 0.05 with olanzapine, < 0.10 with haloperidol, and < 0.05 with chlorpromazine were excluded to produce comparability between groups. To calculate the subgroup-specific HRs and 95% CIs, we stratified the analysis by age group, gender, presence and type of dementia, and comorbidities. We also investigated whether a dose-response relationship existed in adjusted models by separating conventional antipsychotic users into subgroups made up of those taking the median daily dose or less and those taking more than the median daily dose based on the previous research performed by Wang et al. in 2005 [29]. For dose analysis, we used the median daily doses instead of the maximum doses for calculation because median daily doses represent the actual usage more accurately than the maximum doses in cases of dose reductions due to adverse reactions and intermittent medicine use. In addition, our purpose was to evaluate the influence of an increased dose on the risk, and not to compare the absolute risk with specific doses between antipsychotics in a dose-response analysis, so dose equivalencies were not considered [30].

For the a priori calculation of sample size, we calculated the sample size based on the equation of the Cox proportional hazards model using PASS version 12 (NCSS; Kaysville, Utah, USA). The number of patients needed was 30,207. Additionally, we calculated the statistical power to evaluate the appropriateness of the number of study subjects. All of the statistical analyses were performed with SAS version 9.3 (SAS Institute, Cary, NC). A two-tailed value of P<0.05 was considered statistically significant.

### Ethics statement

This study was approved by the Institutional Review Board of Seoul National University College of Medicine/Seoul National University Hospital and Korea HIRA Medical Information Disclosure Committee. Obtaining informed consent from the study population was waived by the board.

## Results

A total of 71,584 patients were included in the cohort, including 24,668 patients treated with risperidone, 15,860 patients with quetiapine, 3,888 patients with olanzapine, 19,564 patients with haloperidol, and 7,604 patients with chlorpromazine. The general characteristics of the patients are shown in Table 1. Patients receiving chlorpromazine were a smaller proportion of the female elderly, had a higher Charlson comorbidity score, were less likely to have dementia, and were more likely to have COPD than other patients receiving AAPs (Table 1).

The incidence rate was higher for those on chlorpromazine (8.01) and haloperidol (6.12) among the CAPs compared to those on quetiapine (4.23), olanzapine (2.87), and risperidone (3.02) among the AAPs per 1,000 person-years. The multivariable adjusted HR for age, gender, presence or absence of dementia, depression, dyslipidemia, coronary heart disease, and the use of antidepressants, benzodiazepines, anticoagulants, and antithrombotic agents during follow-up was estimated. Substantially increased risk was observed for chlorpromazine (HR = 3.10, 95% CI, 1.48–5.11) and haloperidol (HR = 2.09, 95% CI, 1.09–3.12) (Table 2). Among the total study subjects, 99.8% (n = 71,458) were censored. Most of the censoring occurred by discontinuation (n = 57,810, 80.9%), followed by study termination (n = 10,218, 14.3%), and switching (n = 408, 4.7%). No incident cases of ischemic stroke were identified in those patients who switched their antipsychotic drugs.

**Table 2:** Incidence rates and hazard ratios of ischemic stroke after the initiation of conventional and atypical antipsychotic medications.

						Hazard ratio (95% CI)		
User Status	Mean days of follow-up (SD)	Person-Years	No. of Events	Incidence Rate per 1000 Person-Years*	Unadjusted	Propensity-score adjusted	SMR weighed	SMR weighted and Multivariable adjusted†
**Class**								
Atypical antipsychotics(n = 444,16)	150.9(172.6)	18,350.7	62	3.42	1 [Reference]	1 [Reference]	1 [Reference]	1 [Reference]
Conventional antipsychotics(n = 27.168)	130.3 (153.6)	9,690.4	64	6.61	2.18(1.84–2.59)	2.71(2.01–3.52)	2.69 (2.08–3.47)	2.53 (1.95–3.27)
**Generic name**								
Risperidone (n = 24,668)	152.7(167.2)	10,309.4	31	3.02	1 [Reference]	1 [Reference]	1 [Reference]	1 [Reference]
Quetiapine (n = 15,860)	143.9 (172.6)	6,246.2	26	4.23	1.33 (0.81–2.25)	1.35 (0.80–2.27)	1.23 (0.78–2.12)	1.01 (0.61–2.09)
Olanzapine (n = 3,888)	168.6 (202.3)	1,795.1	5	2.87	1.31 (0.47–3.24)	1.25 (0.53–2.95)	1.12 (0.59–2.75)	1.08 (0.47–2.59)
Haloperidol (n = 19,564)	131.9(151.6)	7,066.1	43	6.12	2.61(1.35–3.37)	2.64 (1.27–3.26)	2.43 (1.18–3.14)	2.09 (1.09–3.12)
Chlorpromazine (n = 7,604)	126.3 (158.3)	2,628.3	21	8.01	3.32 (2.02–6.12)	3.50 (2.17–5.65)	3.47(1.97–5.38)	3.10(1.48–5.11)

(SMR, standardized morbidity ratio)

* (No. of events/total No. of days per 365 days)× 1,000.

† Adjusted for age, gender, presence or absence of dementia (F00-F03, G30, G31.8), depression (F32–33, F34.1, F41.2), dyslipidemia (E78.0), coronary heart disease (I21-I25), COPD (J40-J44, J47), and the use of antidepressants, benzodiazepine, anticoagulants, or antithrombotic agents during the follow-up period.

SMR weighted and multivariable adjusted HR after exclusion of the patients whose propensity score was > 99.99 in quetiapine, < 0.05 in olanzapine, > 0.90 in haloperidol, and < 0.05 in chlorpromazine.

**Fig 1:**
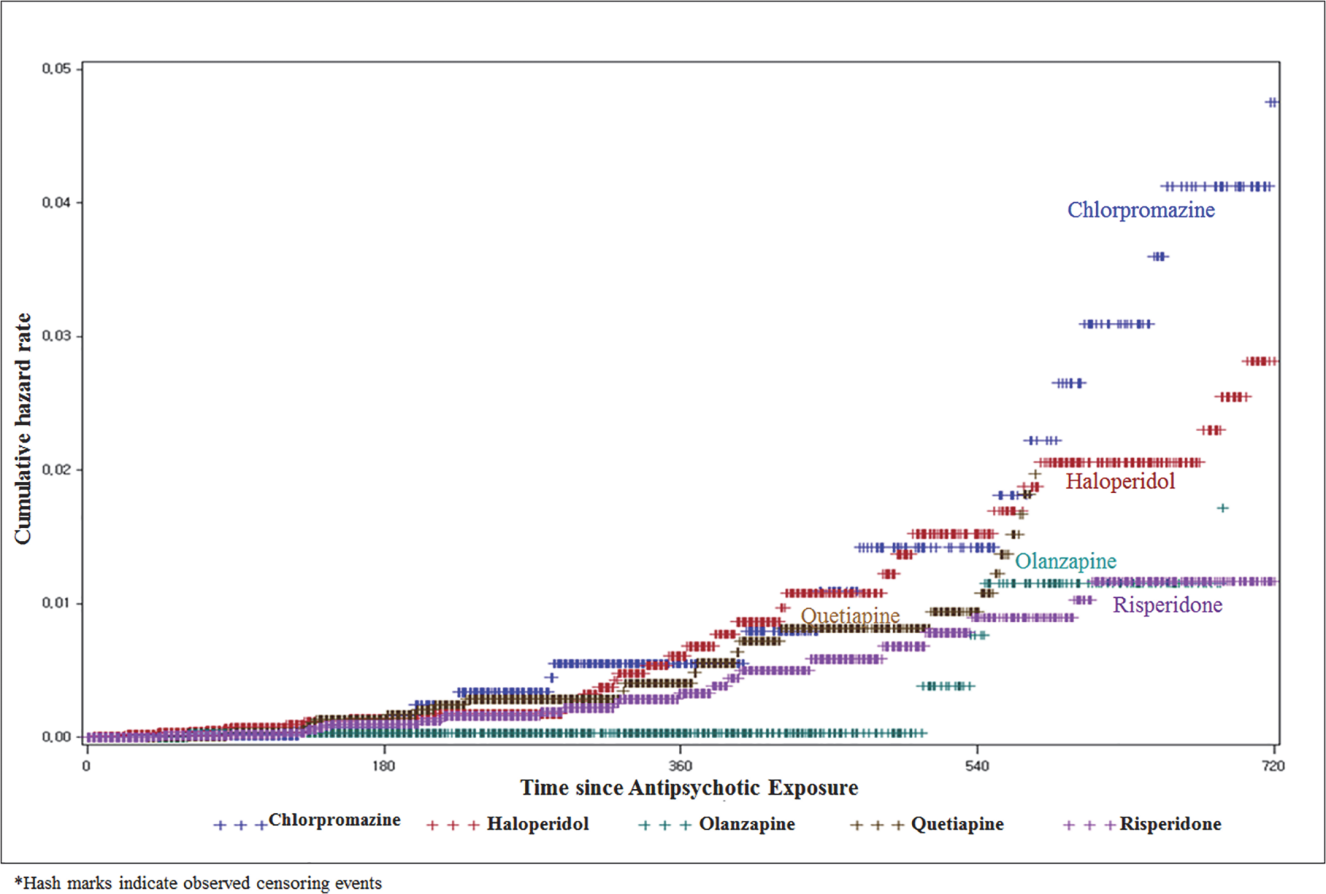
Cumulative hazard rate for ischemic stroke after the initiation of risperidone, quetiapine, olanzapine, haloperidol, and chlorpromazine.

Fig. 1 shows the incidence rates (ischemic stroke per person-year) according to the follow-up days since the initiation of antipsychotic exposure. Consistent with our adjusted models of ischemic stroke, we observed that higher rates of ischemic stroke were associated with CAPs compared with AAPs. Chlorpromazine showed the greatest distinction, followed by haloperidol, and then the other AAPs (Fig. 1).

Chlorpromazine showed a dose-response relationship (p for trend <0.01), whereas haloperidol did not (p for trend = 0.2). After the test for the proportionality assumption, a separate Cox analysis was performed according to the time-varying cross-point. Chlorpromazine showed a higher risk after use for at least 150 days (HR = 3.60, 95% CI, 1.83–6.02), but no significant risk before the use for 150 days (HR = 0.82, 95% CI, 0.43–1.21). Haloperidol showed a similar risk for both periods of use when performing separate analyses of short-term and long-term exposure. C-statistics for each of the models were 0.77 for quetiapine users, 0.88 for the olanzapine users, 0.61 for haloperidol users, and 0.94 for chlorpromazine users compared to the risperidone exposure group (Table 3).

**Table 3:** Dose-response relationship and time-varying risks of ischemic stroke of quetiapine, olanzapine, haloperidol, and chlorpromazine compared with risperidone.

	Quetiapine	Olanzapine	Haloperidol	Chlorpromazine
	(N = 15,860)	(N = 3,888)	(N = 19,564)	(N = 7,604)
**Total HRs** *	1.01 (0.61–2.09)	1.08 (0.47–2.59)	2.09 (1.09–3.12)	3.10 (1.48–5.11)
**Mean PDD**				
Low	…†	…†	2.45 (1.36–4.25)	…†
Median	0.16 (0.06–0.43)	0.27 (0.01–9.11)	2.39 (1.32–4.25)	1.63 (0.94–2.81)
High	2.87 (1.37–6.02)	1.79 (0.26–12.25)	1.86 (1.01–3.43)	3.95 (2.05–7.62)
P for trend‡	<0.01	0.78	0.2	<0.01
**Time interval** ‡				
Short-term‡	**≦90days**	**≦150days**	**≦150days**	**≦150days**
	1.10 (0.72–1.48)	0.53 (0.002–1.06)	1.82 (1.61–2.03)	0.82 (0.43–1.21)
Long-term§	**>90days**	**>150days**	**>150days**	**>150days**
	1.13 (0.44–3.01)	0.55 (0.22–3.21)	2.18 (1.44–3.89)	3.60 (1.83–6.02)

(Mean PDD, mean Prescribed Daily Dose)

*Adjusted for age, gender, presence or absence of dementia (F00-F03, G30, G31.8), depression (F32–33, F34.1, F41.2), dyslipidemia (E78.0), coronary heart disease (I21-I25), COPD (J40-J44, J47), and the use of antidepressants, benzodiazepine, anticoagulants, or antithrombotic agents during the follow-up period.

The estimated HRs were finally accepted as the SMR weighted and multivariable adjusted HR after exclusion of the patients whose propensity score is > 99.99 in quetiapine, < 0.05 in olanzapine, >0.90 in haloperidol, and < 0.05 in chlorpromazine

†Could not be estimated.

‡P for trend was calculated using the likelihood ratio test.

§Short- and long-term periods were distinguished by the cross-point using a log-log survival curve.

Age group-specific, gender-specific, and disease-specific HRs are shown in Table 4. Chlorpromazine showed a higher risk in the older elderly (p for trend<0.01); female patients (p for interaction<0.01); demented patients (p for interaction<0.01); and patients with hypertension, diabetes, or COPD (p for interaction<0.05).

**Table 4:** Sub-group analysis of the risk of ischemic stroke with quetiapine, olanzapine, haloperidol, and chlorpromazine compared with risperidone according to the age group, gender, the presence and type of dementia, and comorbidity.

	Quetiapine	Olanzapine	Haloperidol	Chlorpromazine
	(N = 15,860)	(N = 3,888)	(N = 19,564)	(N = 7,604)
**Total HRs** *	1.01 (0.61–2.09)	1.08 (0.47–2.59)	2.09 (1.09–3.12)	3.10 (1.48–5.11)
**Age, years**				
65–74	0.67 (0.27–1.66)	…*	2.13 (1.38–3.29)	2.13 (1.24–3.66)
75–84	2.43 (1.13–5.22)	3.07 (0.58–16.35)	2.09 (1.15–3.78)	7.55 (4.23–13.48)
85+	1.25 (0.22–7.23)	…*	1.64 (0.57–4.77)	…*
P for trend†	0.78	…*	0.56	<0.01
**Gender**				
Male	1.50 (0.60–3.79)	0.86 (0.04–20.22)	2.09(1.18–3.70)	2.66 (1.60–4.45)
Female	1.04 (0.53–2.04)	0.71 (0.10–5.11)	2.01 (1.34–3.01)	3.98 (2.25–7.03)
P for interaction	0.56	0.69	0.73	<0.01
**The presence of dementia**				
Yes	0.61 (0.29–1.29)	0.75 (0.13–4.28)	2.40 (1.39–4.14)	5.26 (3.48–7.95)
No	2.21 (1.00–4.89)	0.63 (0.013–6.44)	1.70 (1.14–2.52)	2.01 (0.93–4.33)
P for interaction	0.21	0.76	0.06	<0.01
**The presence of hypertension**				
Yes	1.86 (0.29–9.21)	0.85 (0.09–5.28)	2.41 (1.01–5.02)	5.04 (2.48–7.95)
No	0.78 (0.12–7.44)	0.61 (0.01–6.98)	1.95 (1.98–4.21)	1.74 (0.89–3.99)
P for interaction	0.57	0.42	0.49	<0.01
**The presence of diabetes**				
Yes	1.20 (0.31–4.21)	…*	2.98 (1.21–4.87)	4.89 (2.48–7.95)
No	1.01 (1.21–4.55)	1.23 (0.31–9.21)	1.50 (1.07–2.59)	1.87 (0.93–3.91)
P for interaction	0.57	…*	0.06	<0.01
**The presence of COPD**				
Yes	0.98 (0.49–5.21)	0.74 (0.23–5.01)	2.51 (1.49–6.14)	4.21 (3.48–7.95)
No	1.32 (0.78–6.17)	0.67 (0.02–6.9)	2.01 (0.71–3.12)	2.17 (0.93–4.33)
P for interaction	0.63	0.57	0.31	0.04
**The presence of depression**				
Yes	1.21 (0.32–6.22)	1.99 (0.09–9.89)	2.10 (1.41–4.14)	3.12 (3.48–7.95)
No	0.98 (0.21–7.21)	…*	1.99 (0.87–4.52)	3.52 (0.93–4.33)
P for interaction	0.69	…*	0.27	0.54

Adjusted for age, gender, presence or absence of dementia (F00-F03, G30, G31.8), depression (F32–33, F34.1, F41.2), dyslipidemia (E78.0), coronary heart disease (I21-I25), COPD (J40-J44, J47), and the use of antidepressants, benzodiazepine, anticoagulants, or antithrombotic agents during the follow-up period.

The estimated HRs were finally accepted as the SMR weighted and multivariable adjusted HR after exclusion of the patients whose propensity score is > 99.99 in quetiapine, < 0.05 in olanzapine, > 0.90 in haloperidol, and < 0.05 in chlorpromazine

* Could not be estimated.

† P for trend was calculated using likelihood ratio test.

## Discussion

This population-based cohort study revealed a very strong risk of ischemic stroke associated with chlorpromazine and haloperidol compared to risperidone. The evidence showed that AAPs may be preferred to CAPs in elderly patients based on the improved safety profile. Chlorpromazine showed a tripling of the ischemic stroke risk among long-term users (more than 150 days). Of particular note, female and demented patients showed an almost 5-fold increased risk with chlorpromazine compared with risperidone use.

Our results suggest an increased risk of ischemic stroke related to the use of chlorpromazine or haloperidol compared with risperidone. This finding is consistent with previous retrospective studies and adds to the evidence. A previous population-based case-control study in elderly patients showed a 1.83-fold (95% CI, 1.57–2.14) increased rate of cerebrovascular accidents after the use of conventional antipsychotics compared with atypicals. [31] Chlorpromazine and haloperidol were both included in the conventional antipsychotics group in the study, but the risk of individual drugs was not shown. Another retrospective cohort study in the elderly patients with dementia reported that the odds ratio of incident cerebrovascular events for haloperidol was 1.91 (95% CI, 1.02–3.60) compared with risperidone. [32] The estimated odds ratio reported in Finkel et al.[32] was very similar to our estimated hazard ratio for haloperidol (HR = 2.09, 95% CI, 1.09–3.12), and the CIs from our study included the point estimate from the Finkel analysis.

On the other hand, conflicting findings have also been reported. A retrospective cohort study using a Canadian claims database showed no significantly increased risk of ischemic stroke with atypicals compared with conventional drugs [11, 33]. However, that cohort study had a small sample size with insufficient power. Additionally, a self-controlled case-series study in 2008 showed conflicting findings, that the risk of stroke might be higher in patients receiving atypical antipsychotics than in those receiving typical antipsychotics [8]. However, this might be due to the difference in the study design. A self-controlled case-series study does not allow direct comparisons, so it is difficult to conclude that the risk of stroke from atypicals is higher than that from conventional antipsychotics. In addition, Douglas and Smeeth [8] pooled both ischemic and hemorrhagic stroke even though the two events have very distinct mechanisms. Although the results of previous studies have been mixed, our results suggest a higher risk of ischemic stroke in geriatric patients with conventional antipsychotics when compared to atypicals.

It is generally believed that conventional antipsychotics cause more extrapyramidal motor symptoms than atypical antipsychotics, whereas atypical antipsychotics generally cause more weight gain and the metabolic syndrome. [34, 35] However, it should be noted that safety profiles vary for individual antipsychotics. [6, 36] Several studies have reported the difference in mortality risk among antipsychotics. [37–39] Since these studies suggest that the difference in risk of individual drugs may be important, the patient’s risk factors and safety profile of an individual drug should be considered in choosing antipsychotics.

Our results included the comparative risk of ischemic stroke among haloperidol, chlorpromazine, risperidone, quetiapine, and olanzapine. The finding of the highest risk with chlorpromazine is newsworthy, as many of previous studies did not cover chlorpromazine, with one exception. One retrospective cohort study showed that the risk of stroke was six times higher for phenothiazines (the class containing chlorpromazine) and 3.6 times for butyrophenones (the class containing haloperidol) compared with non-users [40]. Our results suggest that prescribing CAPs, particularly chlorpromazine, should be done with more caution in demented female geriatric patients. However, the study results must be interpreted with caution. For example, although higher risk was observed in patients who were prescribed chlorpromazine (HR = 3.10, 95% CI, 1.48–5.11), an immediate discontinuation or drug switch to an atypical is not necessarily required in all cases. When chlorpromazine was used for a shorter period, the risk was only 0.82 (95% CI, 0.43–1.21), and it could be used safely with regular monitoring based on the risk-management guidelines.

Up to now, the biological mechanisms responsible for a possible increased risk of ischemic stroke have remained unknown, although several hypotheses have been suggested [41]. As our study showing that chlorpromazine showed an increased risk in long-term users of more than 150 days, a transient effect may not be involved as a possible mechanism in the case of chlorpromazine. We found a higher risk with conventional antipsychotics compared to atypicals, and a long-term effect may be involved as a possible mechanism. Particularly, differences in safety profiles related to hyperprolactinemia and hypotension among individual antipsychotics have been demonstrated [42]. Hyperprolactinemia has mostly been seen with CAPs, and this implies that drug-induced hyperprolactinemia, which may promote platelet aggregation [43], might be the biological mechanism responsible for increased risk of ischemic stroke. A gender difference in the stratified analysis may also support the hyperprolactinemia hypothesis. Normally, females are more prone to hyperprolactinemia risk than males [42, 44], which may explain our results demonstrating the highest risk in female chlorpromazine users.

This study had several strengths. Our study included the entire Korean population rather than a sample and used the claims database for all antipsychotic users collected by the NHI program, which covered nearly all inhabitants of Korea. This permits a large enough sample size to confirm the hypothesis (power = 83.6%). In addition, we effectively controlled residual confounding using the propensity score and the weighted method. In 2005, the US FDA notified healthcare professionals that patients with dementia-related psychosis treated with atypical antipsychotic drugs are at an increased risk of death. And in 2008, the US FDA noted that both conventional and atypical antipsychotics are associated with the risk. However, these notifications, which were disseminated as “Dear Doctor” letters, were assumed to have influenced the medical practice minimally in our study. A systematic review by Dusetzina et al. demonstrated that many FDA drug risk communications have either delayed or no impact on health behaviors [45]. Despite this general finding, some communications did result in immediate changes in prescribing and patient care. Confounding would occur if chlorpromazine or haloperidol were more likely than atypicals in those who were frailer or at greater risk of ischemic stroke than others. Therefore, using traditional multivariate analysis with propensity score adjustment, SMR weighted analysis was applied, and then the final model was accepted as SMR-weighted and multivariable-adjusted after the exclusion of the patients whose propensity scores were outliers [24]. To reduce the impact of treatment selection bias and potential confounding in an observational study, we performed rigorous adjustment for significant differences in characteristics of patients with the weighted Cox proportional hazards regression models using the standardized morbidity ratio (SMR) [21, 26, 28].

We also provided more detailed information about the timing of stroke using a time-dependent analysis. Previously studies have found higher risk at the beginning of treatment [46, 47]. However, these studies’ control groups were nonusers of antipsychotics, whereas our study compared individual drugs with risperidone. Therefore, our result does not necessarily mean that CAP users are at higher risk of ischemic stroke compared to nonusers. Further research is warranted to demonstrate the timing of stroke in detail. The risk profiles in patients with and without dementia and other comorbidities might be clinically useful.

Our results should be interpreted with caution. The diagnoses reported to HIRA might have differed from the actual diagnoses of patients. In our study, only patients who were diagnosed for ischemic stroke during hospitalization were selected as incident cases. This restriction could have increased the reliability of diagnostic accuracy, and could have lowered the incidence of ischemic stroke compared to other studies. A previous validation study compared the diagnoses derived from the HIRA database with the actual diagnoses recorded in patient medical records. The overall positive predictive value of the diagnoses was 83.4% in cases of hospitalized patients [14, 15]. Another point is that although several potential and measurable risk factors were adjusted for, there may have been unmeasured confounders affecting the results. Even though the claims data represent the whole Korean population, the use of administrative data that lacks the most relevant risk factors for stroke, such as depressive symptoms, SES, and blood pressure, warrants a careful interpretation of the results. The use of the propensity score could not control for unmeasured confounders in the study. For example, physician-specific prescribing preferences or genetic factors that we could not measure may have influenced the clinical outcomes. This is a universal problem with all observational studies.

## Conclusion

In conclusion, our study confirmed a greater risk of ischemic stroke with chlorpromazine and haloperidol when compared with risperidone in the elderly. It is worth noting that chlorpromazine tripled the risk of ischemic stroke among the long-term users (more than 150 days). Female and demented patients showed an almost 5-fold increased risk compared to risperidone users. The evidence suggests that chlorpromazine should be used with caution in the elderly. The assessment of risk and potential benefit should be performed carefully when selecting antipsychotics for optimal clinical outcomes.

## Supporting Information

S1 Table Propensity score distribution for the quetiapine, olanzapine, haloperidol, and chlorpromazine groups compared to that of the risperidone exposure group: Propensity score medians, interquartile ranges (boxes) and 5^th^ and 95^th^ percentiles (whiskers). (DOCX)
