# Supplementary material for: Risk of Ischemic Stroke Associated with the Use of Antipsychotic Drugs in Elderly Patients: A Retrospective Cohort Study in Korea
Source: PLoS One. 2015 Mar 19;10(3):e0119931. doi: 10.1371/journal.pone.0119931 (PMC4366389; doi:10.1371/journal.pone.0119931)
Supplement: S1 Table — (DOCX) [file pone.0119931.s001.docx]

**S1 Table. Propensity score distribution for the quetiapine, olanzapine, haloperidol, and chlorpromazine groups compared to that of the risperidone exposure group:**

**Propensity score medians, interquartile ranges (boxes) and 5^th^ and 95^th^ percentiles (whiskers)**

**
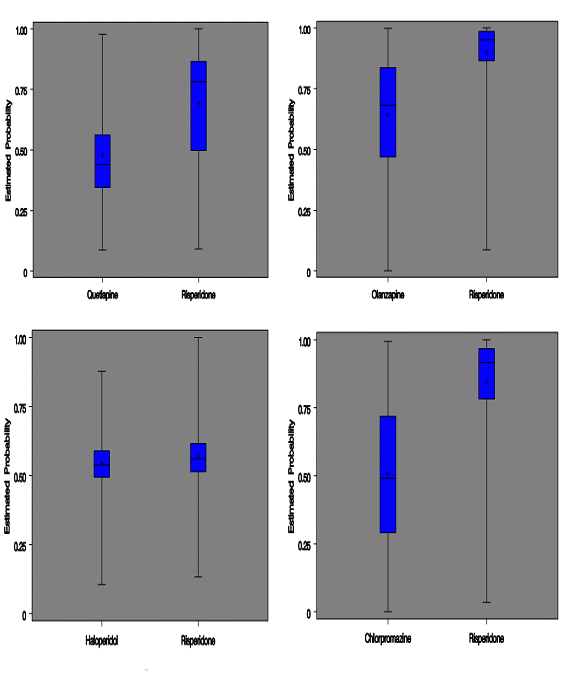
**

*Note: Vertical axis=log10 (propensity score), Horizontal axis=0 for quetiapine, olanzapine, haloperidol, and chlorpromazine group (left), 1 for risperidone group (right).

† C-statistics for each of the models were 0.77 for the quetiapine users, 0.88 for the olanzapine users, 0.61 for the haloperidol users, and 0.94 for the chlorpromazine users compared to the risperidone exposure group.
